# Lesion volume and spike frequency on EEG impact perfusion values in focal cortical dysplasia: a pediatric arterial spin labeling study

**DOI:** 10.1038/s41598-024-58352-9

**Published:** 2024-03-31

**Authors:** Antonio Giulio Gennari, Giulio Bicciato, Santo Pietro Lo Biundo, Raimund Kottke, Ilona Stefanos-Yakoub, Dorottya Cserpan, Ruth O’Gorman Tuura, Georgia Ramantani

**Affiliations:** 1 Department of Neuropediatrics, University Children’s Hospital Zurich, 75, 8032 Zurich, Switzerland; 2 MR-Research Centre, University Children’s Hospital Zurich, Zurich, Switzerland; 3 Department of Neurology, University Hospital Zurich, Zurich, Switzerland; 4 Department of Radiology, University Children’s Hospital Zurich, Zurich, Switzerland; 5 University of Zurich, Zurich, Switzerland; 6 Children’s Research Centre, University Children’s Hospital Zurich, Zurich, Switzerland

**Keywords:** Diagnostic markers, Brain imaging, Magnetic resonance imaging, Paediatric research, Epilepsy

## Abstract

Arterial spin labelling (ASL), an MRI sequence non-invasively imaging brain perfusion, has yielded promising results in the presurgical workup of children with focal cortical dysplasia (FCD)-related epilepsy. However, the interpretation of ASL-derived perfusion patterns remains unclear. Hence, we compared ASL qualitative and quantitative findings to their clinical, EEG, and MRI counterparts. We included children with focal structural epilepsy related to an MRI-detectable FCD who underwent single delay pseudo-continuous ASL. ASL perfusion changes were assessed qualitatively by visual inspection and quantitatively by estimating the asymmetry index (AI). We considered 18 scans from 15 children. 16 of 18 (89%) scans showed FCD-related perfusion changes: 10 were hypoperfused, whereas six were hyperperfused. Nine scans had perfusion changes larger than and seven equal to the FCD extent on anatomical images. Hyperperfusion was associated with frequent interictal spikes on EEG (*p* = 0.047). Perfusion changes in ASL larger than the FCD corresponded to larger lesions (*p* = 0.017). Higher AI values were determined by frequent interictal spikes on EEG (*p* = 0.004). ASL showed FCD-related perfusion changes in most cases. Further, higher spike frequency on EEG may increase ASL changes in affected children. These observations may facilitate the interpretation of ASL findings, improving treatment management, counselling, and prognostication in children with FCD-related epilepsy.

## Introduction

Pharmacoresistance affects approximately 25% of children with epilepsy^1^, and the presence of a brain lesion is its key determinant^2,3^. Malformations of cortical development and, specifically, focal cortical dysplasia (FCD)^4^ constitute the most common substrate of paediatric focal lesional epilepsy^5^. Epilepsy surgery is a safe and effective treatment option for carefully selected children with FCD-associated epilepsy^6–11^ and can potentially offer a cure, i.e., seizure freedom in the absence of antiseizure medication (ASM)^12^, and thus positively impact developmental and cognitive functioning^13–15^. Presurgical evaluation aims to localise the lesion primarily responsible for seizure onset, delineate the epileptogenic zone, and demarcate the eloquent functional regions^12^. Magnetic resonance imaging (MRI) is a central cornerstone of presurgical evaluation, directing the decision to proceed to further non-invasive or invasive exploration and, eventually, to epilepsy surgery^16^. Despite technological advances, FCD may evade MRI detection in roughly one-third of cases, delaying diagnosis and effective treatment and necessitating further investigations^17^. Therefore, second-line techniques^18^ may be required, including less widely available nuclear imaging methods, namely interictal Positron Emission Tomography (PET) or ictal Single Photon Emission Computed Tomography, requiring the intravenous injection of radioactive tracers, exposure to radiation, and additional sedation sessions in younger ages, or invasive electroencephalography (EEG) recordings, entailing risks of haemorrhage, infection, and novel neurological deficit.

Arterial spin labelling (ASL) is an MRI sequence using magnetically labelled water protons in arterial blood to non-invasively image brain perfusion, thus removing the risk associated with contrast medium injection and gadolinium accumulation in human tissues, as required for other perfusion techniques. Despite its commercial availability and recent standardisation for various brain applications^19–21^, ASL, specifically in focal lesional epilepsy, remains a rapidly and widely developing novel field. ASL has recently been shown to increase the diagnostic yield of presurgical evaluation in predominantly paediatric cohorts with neonatal onset hemispheric epilepsy^22^, tuberous sclerosis^23^, and focal lesional epilepsy^24–26^. Although these studies suggested that ASL may be particularly useful for the study of FCD^24^, the most common substrate of paediatric focal lesional epilepsy amenable to surgery^7–9^, current knowledge on FCD, as imaged by ASL, derives mainly from case reports^27,28^, and subsets of larger lesional cohorts^24,25,29^. The only study focusing on paediatric FCD so far included only nine children, all with FCD type IIb, and all but one localised in the frontal lobe^30^. Furthermore, most studies have focused on comparing ASL with other presurgical investigations and surgical outcomes^24,27,29–31^, offering limited insights into the different perfusion patterns encountered in FCD and their correlations with clinical and EEG features. Considering that brain perfusion, captured by ASL, may vary with age^32^ and sedation^33^, these insights are crucial for the interpretation of ASL findings in children with FCD-associated epilepsy, who may profit the most from this new tool.

Therefore, our study aims to close the knowledge gap by evaluating the perfusion changes captured by ASL in children with FCD-related epilepsy and investigating their clinical, EEG, and (anatomical) MRI determinants. We evaluated perfusion images acquired using a single-delay pseudo-continuous ASL sequence qualitatively, visually identifying regional areas of asymmetrical perfusion, and quantitatively, assessing quantitative values derived from cerebral blood flow (CBF) maps.

## Results

### Clinical features

Fifteen children (60% male, Supplementary Fig. 1) with a median age at MRI of 11.5 years (interquartile range, IQR: 2.0–13.7; age < 6 years in 6 cases) and median epilepsy duration of 1.1 years (IQR: 0.8–4.5) were included in our study (Table 1). All patients were MRI-positive; we only considered MRI scans acquired before surgery for our analysis. Seven of 15 (47%) children underwent epilepsy surgery. Histopathology verified FCD I in three cases, FCD II in three cases, and FCD III in one case. Details regarding the lesion characteristics in all patients, including those who underwent epilepsy surgery, are provided in Supplementary Table 1.

**Table 1:** Clinical features, EEG and MRI findings, and histopathology.

CLINICAL FEATURES (n = 15)			
	Male, n (%)		9 (60%)
	Age at MRI in y, median (IQR)		11.5 (2.0–13.7)
	Age at epilepsy onset in y, median (IQR)		1.2 (0.3–5.8)
	Epilepsy duration in y, median (IQR)		1.1 (0.8–4.5)
	Seizure frequency	Seizure free*	5 (33%)
		< 30 seizures	5 (33%)
		≥ 30 seizures	5 (33%)
	History of status epilepticus, n (%)		5 (33%)
	Sedation for MRI acquisition (n = 18), n (%)		10 (56%)
	ASM at MRI (n = 18), n (%)	No ASM	3 (17%)
		One ASM	6 (33%)
		Two or more ASM	9 (50%)
EEG FINDINGS (n = 18)			
	Focal slowing, n (%)		11 (61%)
	Spikes, n (%)		15 (83%)
	Frequent spikes, n (%)		8 (44%)
MRI FINDINGS			
*Anatomical images (n* = *15)*			
	Left lateralisation, n (%)		8 (53%)
	Lobar localisation, n (%)	Frontal	8 (53%)
		Temporal	3 (20%)
		Posterior	3 (20%)
		Multilobar	1 (7%)
*FCD volume (n* = *18)*	Lesion volume in mm^3^, median (IQR)		11,191 (1231–23,078)
*Perfusion images (n* = *18)*			
Perfusion pattern (n = 18)			
	Isoperfused, n (%)		2 (11%)
	Hypoperfused, n (%)		10 (56%)
	Hyperperfused, n (%)		6 (33%)
Perfusion extent (n = 16)			
	Larger, n (%)		9 (56%)
	Equal, n (%)		7 (44%)
HISTOPATHOLOGY (n = 7)			
		FCD I	3 (43%)
		FCD IIa	1 (14%)
		FCD IIb	2 (29%)
		FCD III^$^	1 (14%)

We used per-patient analysis to detail the overall clinical features of the patients, and per-scan analysis to describe the respective EEG and MRI findings. Lobar involvement was classified as frontal, temporal, posterior, and multilobar. Lesions located in the parietal or occipital lobe were grouped as posterior, while lesions defined as multilobar involved more than one lobe, irrespective of the lobes involved.

*n*: Number; *FCD*: Focal cortical dysplasia; *MRI*: Magnetic resonance; *ASM*: Anti-seizure medication; *IQR*: Inter-quartile range; *Posterior*: Involving either the parietal or the occipital lobe; *: Seizure free in the year before the MRI.

All children underwent MRI, including ASL, at one (N = 12) or two (N = 3) different time points due to clinical indication. Thus, 18 MRI scans and the corresponding EEG recordings (seven in wakefulness and 11 both in wakefulness and sleep) were considered for analysis. The median latency between the MRI and EEG acquisition was ± 14 days (IQR: 3–61); seven (39%) of EEGs were acquired within seven days from the MRI.

Ten of 18 (56%) MRI scans were acquired under sedation (Supplementary Table 1). Children who required sedation for MRI acquisition were younger (median: 1.4 years; IQR: 0.6–2.4 years) than children who did not require sedation (median: 13.7 years, IQR: 12.4–15.0 years; *p* < 0.001, Wilcoxon-Mann–Whitney).

### EEG characteristics

The median EEG duration was 87 min (IQR: 46–2156 min) and exceeded 24 h in eight of 18 cases. Focal slowing was detected in 11 of 18 (61%) EEGs. Spikes were detected in 15 of 18 (83%) EEGs; these were frequent in eight cases (Table 1). Focal slowing and spikes colocalised with the FCD in anatomical images and the perfusion changes in ASL in all cases (Supplementary Table 1).

### MRI qualitative analysis

Of 15 FCDs, 8 (53%) were left-sided; eight were frontal, three temporal, three posterior, and one multilobar. Six FCDs were deep-seated: two mesial temporal, one fronto-basal, and three mesial parietal (Table 1). 16 (89%) scans showed perfusion changes ipsilateral to the FCD: 10 were hypoperfused, whereas six were hyperperfused compared to the contralateral brain parenchyma (CBP); nine had perfusion changes larger than the FCD extent, and seven had perfusion changes equal to the FCD extent. In two of three cases scanned twice, the perfusion pattern remained constant, while in one case, the perfusion pattern changed between hyper- and hypoperfused. In all cases with repeat scans, the extent of the perfusion changes remained consistent. The two cases lacking FCD-related perfusion changes did not differ from the other cases as to their clinical, EEG, or anatomical MRI features (Table 2). A hypoperfused pattern was noted in six of ten (60%) scans acquired under sedation and in four of eight (50%) scans acquired without sedation (Supplementary Table 1).

**Table 2:** Hyperperfusion patterns in arterial spin labelling (ASL) are determined by frequent spikes in EEG.

CLINICAL, EEG, AND MRI FEATURES	ASL PERFUSION PATTERNS			*p*-value
	HYPERPERFUSED (n = 6)	ISOPERFUSED (n = 2)	HYPOPERFUSED (n = 10)	
Age at MRI in y, median (IQR)^$^	6.5 (0.7–13.5)	6.7 (-)	6.2 (2.2–12.9)	0.72
Age at epilepsy onset in y, median (IQR)^$^	0.3 (0.3–3.7)	3.1 (-)	2.1 (0.8–6.9)	0.42
Epilepsy duration in y, median (IQR)^$^	1.2 (0.5- 1.8)	3.8 (-)	0.9 (0.5- 1.5)	0.90
Seizure frequency < 30 monthly, %^#^	3 (50%)	1 (50%)	6 (60%)	0.91
History of status epilepticus, %^#^	3 (50%)	0 (0%)	3 (30%)	0.41
Presence of focal slowing, %^#^	3 (50%)	1 (50%)	7 (70%)	0.69
Presence of spikes, %^#^	5 (83%)	2 (100%)	8 (80%)	0.79
Presence of frequent spikes, %^#^	5 (83%)	1 (50%)	2 (20%)	0.047*
FCD volume in mm^3^, median (IQR)^$^	13,144 (3205–22,835)	8785 (-)	11,627 (4310–25,317)	0.87

Perfusion patterns in the ASL are provided in relation to clinical features, EEG, and anatomical MRI findings. y: years; *FCD*: Focal cortical dysplasia; *IQR*: Inter-quartile range; ^#^: Chi-square test; ^$^: Kruskal Wallis test; *Statistical significance.

### MRI quantitative analysis

The median FCD volume was 11,191 mm^3^ (IQR: 4310–23,078 mm^3^).

The mean cerebral blood flow (CBF) values of the FCD (52 ± 15 mL/100 g/min) and the CBP (54 ± 14 mL/100 g/min) did not differ (*p* = 0.97, Welch *t*-test) and did not correlate with age at MRI (Spearman’s correlation: 0.16, *p* = 0.52 and 0.12, *p* = 0.63, respectively). However, CBF values in FCD and CBP were significantly lower under sedation (51 ± 13 and 52 ± 15 mL/100 g/min) than without sedation (57 ± 18 and 56 ± 15 mL/100 g/min; *p* < 0.001, Welch *t*-test). The correspondence between corrected CBF values in the FCD (52 ± 14 mL/100 g/min) and the CBP (54 ± 14 mL/100 g/min) and uncorrected CBF values was within an acceptable range (Supplementary Fig. 2).

Hyperperfusion in ASL was associated with frequent spikes in EEG (*p* = 0.047, Chi-square test), and perfusion changes in ASL larger than the FCD on anatomical images corresponded to larger lesion volumes (*p* = 0.017, Wilcoxon-Mann–Whitney test). No other epilepsy-related or lesion-related features significantly impacted the respective perfusion changes (Tables 2, 3).

**Table 3:** Perfusion changes in arterial spin labelling (ASL) larger than the focal cortical dysplasia (FCD) correspond to smaller lesion volumes.

CLINICAL, EEG, AND MRI FEATURES	ASL PERFUSION EXTENT		*p*-value
	LARGER (n = 9)	EQUAL (n = 7)	
Age at MRI in y, median (IQR)^$^	3.5 (1.3–11.5)	11.6 (2.0–15.3)	0.37
Age at epilepsy onset in y, median (IQR)^$^	2.1 (0.3- 4.8)	0.6 (0.3- 5.5)	0.92
Epilepsy duration in y, median (IQR)^$^	1.0 (0.7–1.3)	1.0 (0.3–1.9)	0.79
Seizure frequency < 30 monthly, %^#^	4 (44%)	5 (71%)	0.57
History of status epilepticus, %^#^	5 (56%)	1 (14%)	0.24
Presence of focal slowing, %^#^	7 (78%)	3 (43%)	0.36
Presence of spikes, %^#^	9 (100%)	4 (57%)	0.13
Presence of frequent spikes, %^#^	4 (44%)	3 (43%)	1
FCD volume in mm^3^, median (IQR)^$^	23,809 (10,798–30,089)	3935 (2965–8944)	0.017*

The extent of perfusion changes in the ASL is provided in relation to clinical features, EEG, and anatomical MRI findings.

*y*: Years; *FCD*: Focal cortical dysplasia; *IQR*: Inter-quartile range; ^#^: Chi-squared test; ^$^: Mann–Whitney test; *Statistical significance.

**Figure 1:**
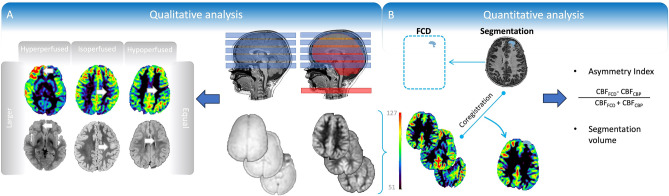
MRI analysis pipeline of anatomical and perfusion images. A. Qualitative analysis. The perfusion changes corresponding to the focal cortical dysplasia (FCD) were characterised as hyperperfused, isoperfused, or hypoperfused compared to the contralateral brain parenchyma (CBP). The extent of the perfusion changes corresponding to the FCD was characterised as “larger” or “equal” compared to the lesion extent in anatomical images. B. Quantitative analysis. Anatomical images (volumetric T1-weighted and FLAIR) were used for lesion segmentation. Segmentations were manually drawn by the same radiologist who performed the qualitative analysis, encompassing the signal intensity changes visible on anatomical images. Both grey and white matter were included in the polygonal, volumetric region of interest (ROI). Dilation-erosion and smoothing algorithms were used to minimise segmentation errors. Before saving the segmentation as a binary label, the ROI position was carefully checked against that of the brain gyri and adjusted as needed. FSL (www.fmrib.ox.ac.uk/fsl) was used to co-register T1-weighted images to ASL by rigid body registration. Subsequently, T1-weighted images were co-registered to the MNI152 atlas, extending the co-registration process to ASL images and binary labels. The perfusion values were computed by extracting the mean cerebral blood flow (CBF) values within the binary labels. The CBF values were first calculated within the binary label deriving from the FCD and then within the flipped label corresponding to the CBP. CBF: cerebral blood flow; CBP: contralateral brain parenchyma; FCD: focal cortical dysplasia.

The mean AI was 0.002 ± 0.13. AI was not associated with age at MRI (Spearman’s correlation: -0.08, *p* = 0.72) and did not differ between scans acquired under sedation (− 0.004 ± 0.1) and those acquired without sedation (0.01 ± 0.1; *p* = 0.84, Welch *t*-test) (Fig. 1).

**Figure 2:**
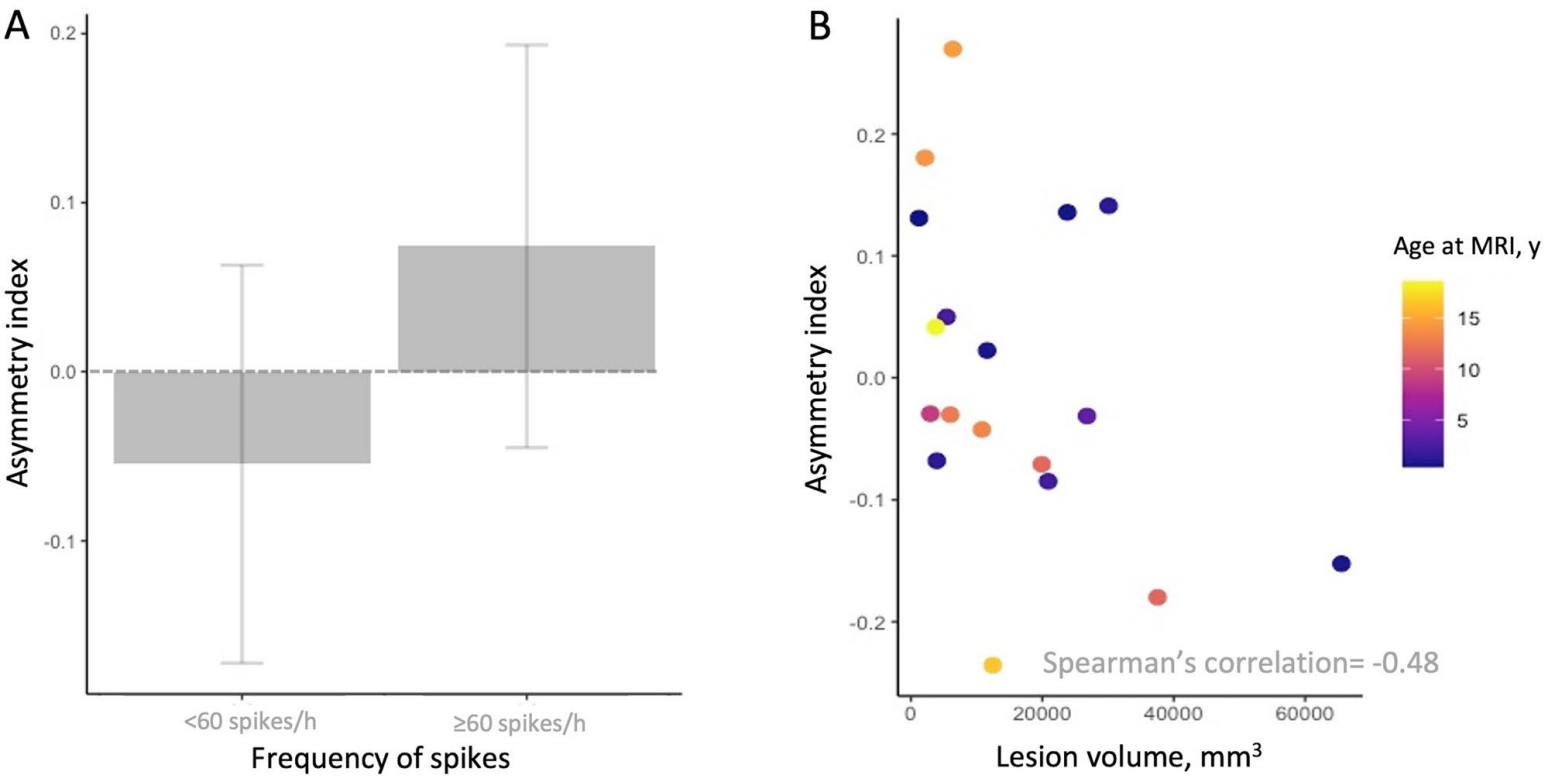
Higher Asymmetry Index (AI) values related to frequent spikes in EEG and by smaller lesion volumes. (A) Higher asymmetry index (AI) values were determined by frequent interictal spikes (≥ 60 spikes/h, n = 8; < 60 spikes/h, n = 10) on electroencephalography (EEG, *p* = 0.004, Welch t-test). The bar plot illustrates the AI values' mean and standard deviation, depending on frequent spikes on interictal EEG. (B) The scatterplot illustrates the correlation between the AI values and the focal cortical dysplasia (FCD) volume when controlled for age at MRI. The data points are colour-coded according to the age at MRI. Smaller FCD volumes showed a statistical trend of being correlated with higher AI values after controlling for age at MRI (Spearman’s correlation: − 0.48, *p* = 0.05). *AI: asymmetry index; EEG:* electroencephalography*; FCD: focal cortical dysplasia; h: hour; y: years.*

Higher AI values were associated with frequent spikes in EEG (*p* = 0.004, Welch *t*-test, Fig. 2A). In the univariate analysis, smaller FCD volumes showed a statistical trend of being correlated with higher AI values after controlling for age at MRI (Spearman’s correlation: − 0.48, *p* = 0.05, Fig. 2B), whereas other epilepsy-related or lesion-related features were not related to AI values (Table 4). Frequent spikes retained their significance as an independent predictor of higher AI values in the multivariate analysis even after correcting for lesion volume (adjusted R^2^: 0.31, *p* = 0.03, Supplementary Table 2).

**Table 4:** Higher Asymmetry Index (AI) values are determined by frequent spikes and smaller focal cortical dysplasia (FCD) volumes.

CLINICAL, EEG, AND MRI FEATURES		ASYMMETRY INDEX	*p*-value
Age at MRI in y^$^		–0.08	0.72
Age at epilepsy onset in y^$^		–0.03	0.90
Epilepsy duration in y^$^		0.22	0.38
Seizure frequency, mean ± SD^#^	< 60 monthly	–0.008 ± 0.13	0.60
	≥ 60 monthly	0.04 ± 0.14	
History of status epilepticus, mean ± SD ^#^	Positive	–0.008 ± 0.13	0.81
	Negative	0.008 ± 0.14	
Presence of focal slowing, mean ± SD ^#^	Positive	0.004 ± 0.11	0.96
	Negative	0.0005 ± 0.17	
Presence of spikes, mean ± SD ^#^	Positive	0.004 ± 0.12	0.95
	Negative	–0.005 ± 0.21	
Presence of frequent spikes, mean ± SD ^#^	Positive	0.07 ± 0.12	0.004*
	Negative	–0.05 ± 0.12	

The AI values are provided in relation to clinical features, EEG and anatomical MRI findings.

*y*: years; *FCD*: Focal cortical dysplasia; *SD*: Standard deviation; ^#^: Welch t-test; ^$^: Spearman’s correlation; *Statistical significance.

## Discussion

To our knowledge, this is the largest study to evaluate the perfusion changes captured by ASL in children with FCD-related epilepsy and to investigate their determinants. ASL showed FCD-related perfusion changes in all but two cases. In the qualitative analysis, the FCD-related hyperperfusion pattern was determined by frequent focal spikes but not by focal slowing in EEG, while larger FCD-related perfusion changes corresponded to larger lesion volumes in anatomical MRI. In the quantitative analysis, higher AI values were associated with frequent spikes after correcting for lesion volume. These observations may facilitate the interpretation of ASL findings in children with FCD-related epilepsy and thus improve treatment management, counselling, and prognostication.

All but two scans showed FCD-related perfusion changes in our study, corroborating previous findings from small-size, predominantly frontal lobe epilepsy case series^24,27,28,30^ in a larger cohort, representative for temporal and extratemporal epilepsy, deep-seated and superficial lesions, and the entire paediatric age range. It should be noted that FCD-related perfusion changes were detectable on ASL despite sedation in half of our cohort, in contrast with the only previous study focusing on ASL in FCD-related epilepsy, which considered exclusively scans performed without sedation^30^. This observation is encouraging, considering the young age at epilepsy onset and the impaired cognitive functioning in children undergoing presurgical evaluation^11,13–15,34–38^—and requiring sedation for MRI acquisition—along with the benefits of early referral and, if indicated, early surgical intervention in this vulnerable population^16^.

FCD-related hyperperfusion**,** observed in one-third of scans in our study, correlated with frequent EEG spikes but not with other epilepsy-related features. This lack of correlation between specific perfusion patterns and clinical or imaging features mirrors a recent PET study in temporal lobe epilepsy^39^. Commonly, hyperperfusion in FCD has been attributed to seizure activity, focally or regionally increasing the CBF^40^. However, hyperperfusion has been shown to persist for days to weeks after the resolution of the ictal state^22,41,42^, particularly after prolonged or repetitive seizures, suggesting a more complex correlation with brain activity. A recent study in pediatric lesional epilepsy linked frequent spikes to hypermetabolic patterns in PET, suggesting that a state of increased regional epileptogenicity may contribute to increased regional metabolism^43^. Interestingly, in this cohort of histopathologically verified FCD-associated epilepsy, only a few patients (13%) had clinical or subclinical seizures during their PET/EEG evaluation^43^. Repetitive epileptic discharges, the characteristic interictal EEG pattern mirroring the high intrinsic epileptogenicity of FCD^44^, may underlie perfusion changes, suggesting an ictal state, increasing the visibility of perfusion changes in ASL. ASL revealed FCD-related hypoperfusion in studies involving older children and adults^29,30^, but FCD-related hyperperfusion in a case series involving infants and toddlers^28^. Although hyperperfusion was not exclusive to the younger patients or earlier stages of the disease in our study, the timing of ASL acquisition may still play a role. Neuronal hyperexcitability may induce functional and metabolic changes leading to CBF increase at epilepsy onset^29^, whereas loss of neurons and gliosis over time may induce neuronal functional and metabolic isolation^45^, as reported in local field potential studies, leading to CBF decrease^46^. This pathophysiological mechanism may also explain the change in perfusion pattern observed in one of three children scanned twice in our study. Finally, it should be noted that irrespective of the perfusion pattern corresponding to the epileptogenic lesion, perfusion changes point to abnormal activity, constituting a valuable indicator of the epileptogenic zone.

FCD-related perfusion changes in ASL more extensive than the anatomical lesions, observed in one-half of scans in our study, correlated with larger lesion volume but not with other epilepsy-related or lesion-related features. This observation is not surprising since larger epileptogenic lesions are intuitively expected to result in a larger functional disruption and, thus, more extensive perfusion changes. However, this finding is encouraging since larger lesions, the trademark of paediatric epilepsy surgery^10,36^, predispose to earlier epilepsy onset and a more severe epilepsy course, were less likely to have ever been reported MRI-negative, and more likely to have undergone epilepsy surgery in a recent large multicentric study^47^. Interestingly, our study is the first to investigate the effect of lesion volume on the visibility of perfusion changes imaged by ASL since previous studies focused mainly on the colocalisation of the perfusion changes with the anatomical lesions at the gyral or lobar scale^24,29,30^. Although the presence of perfusion changes beyond the MRI-visible lesion may correspond to a more subtle—and thus less clearly discernible—part of the cortical malformation, it should be noted that focal lesional epilepsy is a network disease, often involving widespread connections beyond the seizure onset zone and the epileptogenic lesion^45,48,49^. This concept has been previously established in PET studies, where metabolic changes usually extend beyond the epileptogenic lesions, particularly in temporal lobe epilepsy^39^. Perfusion changes may thus be perceived as a marker of the epileptic network, accounting for the stability of ASL findings in children scanned twice in our study. Advanced techniques, such as ASL, expand the potential of MRI beyond mere lesion identification, contributing to the non-invasive delineation of affected brain tissue within the often more extensive epileptic network^45^, supporting hypotheses based on electro-clinical correlations, and guiding successful surgical intervention.

Higher AI values were determined by frequent EEG spikes after correcting for lesion volume, corroborating the results of expert-driven visual analysis by using the data-driven standardised and reproducible AI measure. Of note, a PET study in temporal lobe epilepsy reported a high correlation between the number of interictal spikes recorded in temporal EEG electrodes and the AI values, reflecting the metabolism imaged by PET in the temporopolar regions^50^. Our study corroborates the observation of a link between EEG and brain metabolism, as imaged by PET, and extends it to a link between EEG and brain perfusion, as imaged by ASL, highlighting the opportunities offered by modern imaging tools in the presurgical evaluation of paediatric lesional epilepsy. In this setting, AI offers several advantages over the visual-only evaluation of perfusion maps, contributing to higher FCD-detectability and concordance rates with other tools, such as EEG, MRI, and PET, in detecting the epileptogenic zone^25^. By facilitating the standardisation of perfusion values across patients, AI can counteract the effects of other potential confounders, such as age or sedation, as evidenced by our study. Thus, voxel-wise approaches of AI calculation highlighting perfusion asymmetries between the hemispheres have been increasingly implemented in the latest ASL studies^25,31^ to obtain observer-independent results and increase confidence in image reporting. The recent observation that specific interictal spike patterns in presurgical EEG are predictive of postsurgical seizure freedom in FCD-associated epilepsy^51^, gives additional weight to the link between ASL and interictal spikes substantiated for the first time in our study, pointing to exciting avenues of ASL application beyond the obvious benefits for image reporting and lesion detection.

Although EEG simultaneous to the ASL acquisition should facilitate the differentiation between interictal and ictal ASL findings, this setup has been available in only one small-size study so far and has added no value over other presurgical modalities with less time-consuming recording and analysis^31^. Despite these sparse and disappointing results, EEG-guided image analysis may still add to the diagnostic value of each modality alone by aligning ASL perfusion changes with EEG signal modifications and enabling their interpretation.

Future studies in larger and homogeneous paediatric FCD-related epilepsy cohorts are expected to shed some light on the ASL features and their potential in the particularly challenging subgroup of MRI-negative cases. In contrast to the overall poor yield of visual ASL analysis in MRI-negative FCD-related epilepsy^24,25^, the increasingly implemented quantitative ASL analysis has so far shown promising results^25,31^. It remains to be investigated if the findings derived from our MRI-positive FCD cohort are applicable to MRI-negative cohorts.

Considering that the spiking rate in EEG increases in sleep, particularly in the N2 and N3 sleep stages, in the majority of focal epilepsy patients^52^, long-term EEG recordings capturing these sleep stages^45^ are more suitable to investigate the correlation of frequent spikes on EEG with perfusion changes on MRI. The availability of longer data segments including all vigilance states across all patients would help overcome the limitations of our study, partly drawing from shorter recordings, and offer a more detailed insight into this intriguing correlation of epileptic activity and brain perfusion.

## Conclusion

Our study confirmed that most FCD generate perfusion changes on ASL images. Hyperperfused FCD correlates with a higher spike rate, likely reflecting a higher degree of epileptogenicity. Moreover, larger lesions commonly induce more extensive perfusion changes, mirroring a more extensive epileptogenic network. Finally, higher AI values also relate to higher spike rates in EEG. In addition to facilitating the interpretation of ASL findings, connecting interictal epileptic activity with brain perfusion opens promising perspectives for the future clinical and research implementation of ASL in paediatric epilepsy studies. In the clinical setting, our observations foster the introduction of ASL in imaging protocols, potentially improving treatment management, counselling, and prognostication in children with FCD-related epilepsy. In the research field, ASL findings may provide a new biomarker in this patient subgroup and a valid addition to automatic FCD detection tools.

## Materials and methods

### Patient selection

We included children and adolescents who underwent brain MRI between January 2017 and March 2023 at the University Children’s Hospital Zurich, according to our dedicated epilepsy protocol^10,15,34,36,53–55^. The inclusion criteria were: (1) age ≤ 19 years at the time of the MRI, (2) diagnosis of focal structural epilepsy, according to seizure semiology, EEG, and MRI findings, (3) diagnosis of FCD according to radiological criteria^56,57^, corroborated by histopathology in surgical cases^58^, (4) no history of prior resective epilepsy surgery, and (5) availability of ASL MRI sequences. We excluded MRI scans with overall poor quality (Supplementary Fig. 1). The collection of patient data and their analysis were approved by and performed according to the guidelines and regulations of the local ethics committee (Kantonale Ethikkommission Zürich, KEK-ZH 2024-00298). Informed consent was obtained from all subjects and/or their legal guardian(s).

### EEG acquisition and analysis

EEGs were acquired with 21 electrodes placed according to the international 10–20 system by the Micromed® (Mogliano Veneto, Treviso, Italy) or Deltamed® (Paris, France) recording systems. The EEG concurrent to each MRI was visually reviewed by a fully trained EEG technician and a fully trained neurologist, blinded to the clinical report. Discrepancies were resolved by consensus. EEGs in wakefulness and/or sleep were analysed depending on their availability. Sleep staging was performed according to the American Academy of Sleep Medicine (AASM) guidelines^59^. The presence of focal slowing and spikes was noted. These parameters were selected based on previous work suggesting a correlation between *focal slowing* and ASL perfusion changes in tuberous sclerosis^60^, and between *spikes* and ASL perfusion changes in temporal lobe epilepsy^61^. Spike *frequency* was calculated considering all spikes noted in the first hour of wakefulness and in the first hour of non-rapid eye movement (NREM) sleep, where applicable^62^ and dichotomized^63^ in “frequent” at ≥ 60 spikes per hour and “non-frequent” at < 60 spikes per hour.

### MRI acquisition

All MRI scans were acquired on a 3 T scanner (Discovery MR 750 or Signa Premier, GE Medical Systems, WI, USA), using an 8-channel coil^34,54,55^. Anatomical and perfusion images (ASL) were acquired in all cases. Parameters for anatomical image acquisition have been provided in our previous work^53^, while those for ASL acquisition were: pseudo-continuous ASL, echo time 10.5–11.2 ms, repetition time 4531–4742 ms, single post labelling delay of 1525 ms, flip angle 111°, averages 3, slice thickness 4 mm, spacing between slices 4 mm, and spiral readout. Quantitative perfusion maps were automatically generated by GE reconstruction software. Children underwent sedation if clinically indicated^26^. Since no clinical seizures were noted during the MRI scan, the perfusion patterns shown by the scans were considered to mirror the brain perfusion during the interictal state.

### MRI qualitative analysis

Anatomical and perfusion images were retrieved from the picture archive and communication system and reviewed by a fully trained radiologist with 7 years of experience in neuroradiology.

Anatomical images were used to define the lateralisation, lobar localisation, and extent of the FCDs.

Perfusion images were visually inspected for differences between hemispheres and compared with anatomical images to identify regional perfusion changes corresponding to the FCDs. Perfusion changes within the FCD visible in at least two consecutive slices^25^ were characterised as hypoperfused, isoperfused, or hyperperfused compared to the CBP (Fig. 1). The comparison between the visually assessed extent of the perfusion changes on perfusion images (irrespective of the perfusion pattern), and the extent of the FCD on anatomical images was dichotomised into “equal” (i.e., the extent of perfusion changes matched the extent of the lesion on anatomical images) or “larger” (i.e., the extent of perfusion changes exceeded the extent of the FCD on anatomical images).

### MRI quantitative analysis

Slicer (https://www.slicer.org) and FSL (www. fmrib.ox.ac.uk/fsl) were used to quantify the perfusion changes within FCD and CBP. Post-processing steps are detailed in Fig. 1. The asymmetry index (AI) was used to normalise the automatically generated CBF values for each MRI and across different scans, allowing the identification of asymmetries between cerebral hemispheres and their lobes^64^. The AI was calculated as follows:

$$AI= \frac{CB{F}_{FCD}-CB{F}_{CBP}}{CB{F}_{FCD}+CB{F}_{CBP}}$$

### Statistical analysis

Absolute numbers and percentages were used to report dichotomic and categorical variables, while median and IQR or mean ± standard deviation (SD) were used to report continuous variables. Shapiro–Wilk test was used to test for normality. Chi-Square and Fisher’s exact tests were applied to compare the distribution of categorical variables. Group-wise comparison of continuous variables was performed using two-tailed *non-parametric* (non-normally distributed variables) or *parametric tests* (normally distributed variables). Correlation between variables was tested using Spearman’s correlation coefficient.

Since the CBF values of the brain vary during childhood, Spearman’s correlation was used to investigate the impact of age at MRI on CBF in FCD and CBP^32^. The correlation between FCD volume and AI values was explored by applying Spearman's correlation while correcting for age at MRI^32^. Welch’s *t*-test was used to study the effect of sedation on CBF values calculated within the FCD and the CBP^33^. To evaluate the reliability of the automatically generated CBF values, a sensitivity analysis was performed. Since the ASL signal depends on the T1 relaxation time of blood, which strictly correlates with the blood haematocrit (Hct)^65^, normative Hct values depending on age at MRI were used to generate corrected CBF values, which were then compared with automatically generated CBF values using Bland–Altman plots.

The AI values, which showed the highest correlation between perfusion and EEG findings at univariate analysis (specifically spike rate), were subsequently entered into a multivariable regression model aiming to correct the results for possible covariates, specifically lesion volume. The latter was selected among the MRI-based parameters, showing the highest correlation with ASL findings.

The statistical analysis was performed using* R* software (https://www.r-project.org) version *4.0.5*. Statistical significance was set at *p* < 0.05; Holm’s correction was used for multiple comparisons.

### Ethical approval

The collection of patient data and their analysis were approved by and performed according to the guidelines and regulations of the local ethics committee (Kantonale Ethikkommission Zürich, KEK-ZH 2024-00298). Informed consent was obtained from all subjects and/or their legal guardian(s).

### Supplementary Information

Supplementary Information.

## Data Availability

The data supporting this study's findings are available from the corresponding author upon reasonable request.
