# Supplementary material for: Lesion volume and spike frequency on EEG impact perfusion values in focal cortical dysplasia: a pediatric arterial spin labeling study
Source: Sci Rep. 2024 Mar 31;14:7601. doi: 10.1038/s41598-024-58352-9 (PMC10982306; doi:10.1038/s41598-024-58352-9)
Supplement: Supplementary file 1 — Supplementary Information. [file 41598_2024_58352_MOESM1_ESM.pdf]

LESION VOLUME AND SPIKE FREQUENCY ON EEG IMPACT PERFUSION VALUES IN FOCAL CORTICAL DYSPLASIA: A PEDIATRIC ARTERIAL SPIN LABELING STUDY

Antonio Giulio Gennari[1,2], Giulio Bicciato[1,3], Santo Pietro Lo Biundo[1], Raimund Kottke[4], Ilona Stefanos-Yakoub[1], Dorottya Cserpan[1], Ruth O'Gorman Tuura[2,5,6,$], Georgia Ramantani[1,5,6,$,*]

[1] Department of Neuropediatrics, University Children's Hospital Zurich, Switzerland; [2] MR-Research Centre, University Children's Hospital Zurich, Switzerland; [3] Department of Neurology, University Hospital Zurich, Switzerland; [4] Department of Radiology, University Children's Hospital Zurich, Switzerland; [5] University of Zurich, Switzerland; [6] Children's Research Centre, University Children's Hospital Zurich, Switzerland
[$]: These authors contributed equally to this work.

**Corresponding Author*: Prof. Georgia Ramantani, MD, PhD; Department of Neuropediatrics; University Children's Hospital Steinwiesstrasse 75, 8032 Zurich, Switzerland; phone: +41 44 266 75 92; Email: georgia.ramantani@kispi.uzh.ch

Supplementary Table 1. Clinical features, EEG and MRI findings, need for sedation, and anaesthetics used for sedation.

Pat. Nr.	Sex	Age at epilepsy onset, y	Age at MRI, y	Seizure frequency, monthly	History of status epilepticus	Focal slowing in EEG	Spikes in EEG	Frequent spikes in EEG	Sedation for MRI	Anaesthetics for sedation	Lobar localization	ASMs at scan	Lesion characteristics	Lesion volume, mm^3	ASL perfusion pattern	ASL perfusion extent	Resective surgery	FCD type[#]
1	M	0.3	0.3	>30 seiz.	No	Yes	Yes	Yes	Yes	Ket., Prop.	Frontal	None	CT, GWMb	1231	Hyperperfused	Equal	Yes	IIb
2	F	0.4	0.6	>30 seiz.	No	Yes	Yes	Yes	Yes	Mida., Prop.	Temporal	VPA, TPM	CT, GWMb	11584	Isoperfused	-	Yes	III
3	F	0.2	0.5	>30 seiz.	Yes	Yes	Yes	Yes	Yes	Ket., Prop.	Temporal	OXC, VGB	CT, GWMb	23809	Hyperperfused	Larger	Yes	IIa
4	M	0.3	0.7	>30 seiz.	No	Yes	Yes	No	Yes	Ket.; Prop.	Frontal	VGB, VPA, OXC, CORTI	CT, GWMb	65534	Hypoperfused	Larger	Yes	I
3[#]	F	0.2	1.3	>30 seiz.	Yes	Yes	Yes	Yes	Yes	Ket., Prop.	Temporal	VPA, TPM	CT, GWMb	30089	Hyperperfused	Larger	Yes	IIa
5	F	1.2	1.4	≤30 seiz.	No	No	Yes	No	Yes	Ket., Prop., Nlp	Frontal	LEV	GWMb, TMS	3935	Hypoperfused	Equal	Yes	-
1[#]	M	0.3	2.5	>30 seiz.	No	Yes	Yes	Yes	Yes	Prop.	Frontal	OXC, VPA, CLB	CT, GWMb	5433	Hypoperfused	Equal	Yes	IIb
6	F	2.1	2.1	>30 seiz.	No	Yes	Yes	Yes	Yes	Ket., Prop.	Frontal	None	GWMb	20885	Hypoperfused	Larger	No	I
6[#]	F		3.5	>30 seiz.	No	Yes	Yes	No	Yes	Ket., Prop.	Frontal	OXC, VPA, STM	GWMb	26794	Hypoperfused	Larger	Yes	I
7	M	8.7	8.9	≤30 seiz.	Yes	Yes	Yes	No	No	No sedation	Posterior	LEV, CORTI	CT, GWMb	2969	Hypoperfused	Larger	No	-
8	F	3.3	11.5	≤30 seiz.	Yes	Yes	Yes	No	Yes	Prop.	Temporal	LTG	CT, GWMb	37542	Hypoperfused	Larger	Yes	IIb
9	M	0.3	11.6	seiz. free	Yes	No	Yes	Yes	No	No sedation	Multilobar	LEV	CT, GWMb, TMS	19911	Hyperperfused	Equal	No	-
10	M	5.8	12.7	≤30 seiz.	No	No	Yes	No	No	No sedation	Frontal	STM, LTG	CT, GWMb	5986	Isoperfused	-	Yes	I
11	M	8.1	13.3	seiz. free	Yes	No	Yes	No	No	No sedation	Frontal	None	CT	10798	Hypoperfused	Larger	No	-
12	M	4.8	14.5	seiz. free	No	No	Yes	Yes	No	No sedation	Frontal	OXC, LEV	GWMb	6376	Hyperperfused	Larger	No	-
13	M	14.7	14.1	≤30 seiz.	No	No	No	No	No	No sedation	Posterior	VPA	GWMb	2148	Hyperperfused	Equal	No	-
14	M	0.6	16.5	seiz. free	No	No	No	No	No	No sedation	Frontal	CBZ	CT, TMS	12455	Hypoperfused	Equal	No	-
15	F	9.7	18.7	seiz. free	No	Yes	No	No	No	No sedation	Posterior	LTG	CT, GWMb	3781	Hypoperfused	Equal	No	-

Pat: patient; nr: number; [#]: repeated scans; M: male; F: female; y: years; seiz.: seizure; FCD: focal cortical dysplasia; Ket.: ketamine; Prop.: propofol; Nlp: nalbuphine; Mida: midazolam; CT: cortical thickening; GWMb: blurring of the grey-to-white matter junction; TMS: transmantle sign; ASM: anti-seizure medication; VPA: valproate; TPM: topiramate; OXC: oxcarbazepine; VGB: vigabatrin; CORT: hydrocortisone; LEV: levetiracetam; CLB: clobazam; STM: sultiam; LTG: lamotrigine; CBZ: carbamazepine; ASL: arterial spin labelling; [#]: based on histopathology

Supplementary Table 2. Higher AI values are determined by frequent spikes, independent of FCD volume.
Multivariate linear regression model exploring the relation between AI and spike frequency, corrected for lesion volume.

	**Estimate**	**T value**	***p*-value**
**Intercept**	-2.9×10^{-03}	-0.064	0.03*
**Presence of frequent spikes**	1.2×10^{-01}	2.33	0.03*
**FCD volume**	-3.0×10^{-06}	-1.84	0.09

Supplementary Figure 1. Patient and data selection

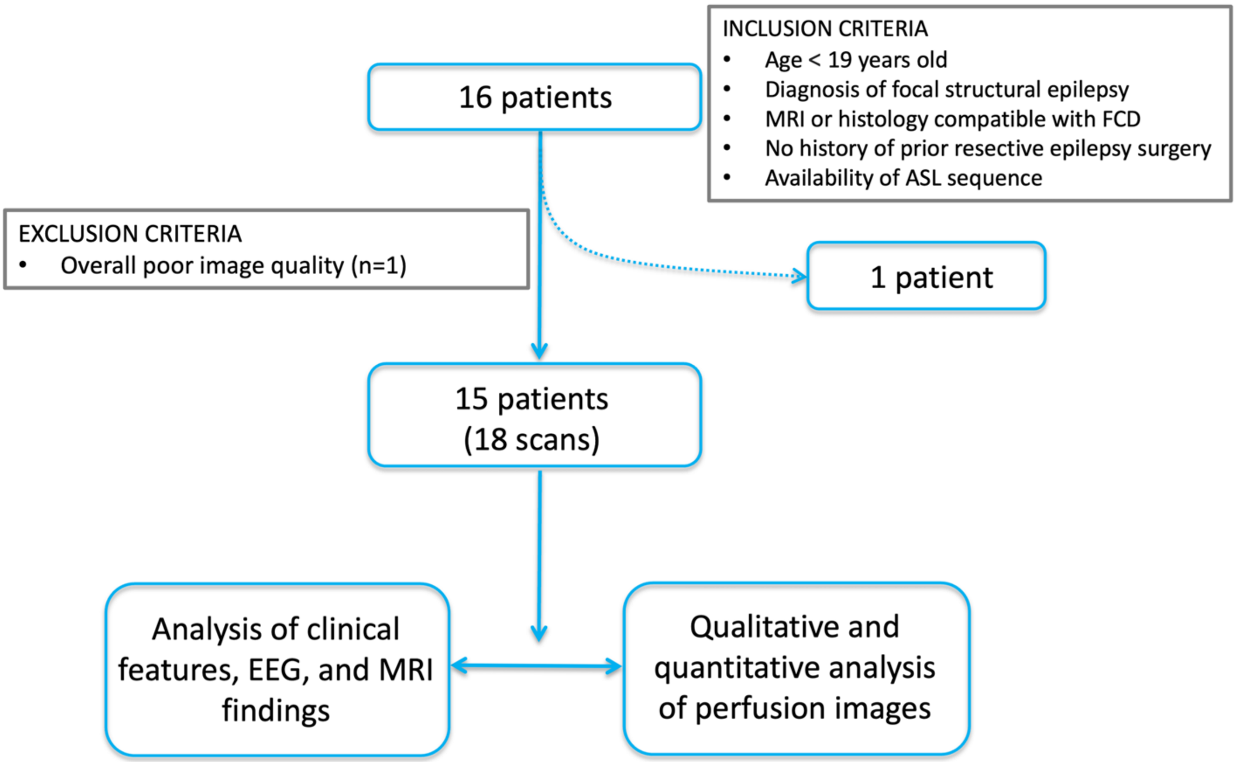

Supplementary Figure 2. Agreement between cerebral blood flow (CBF) values and corrected haematocrit corrected CBF values

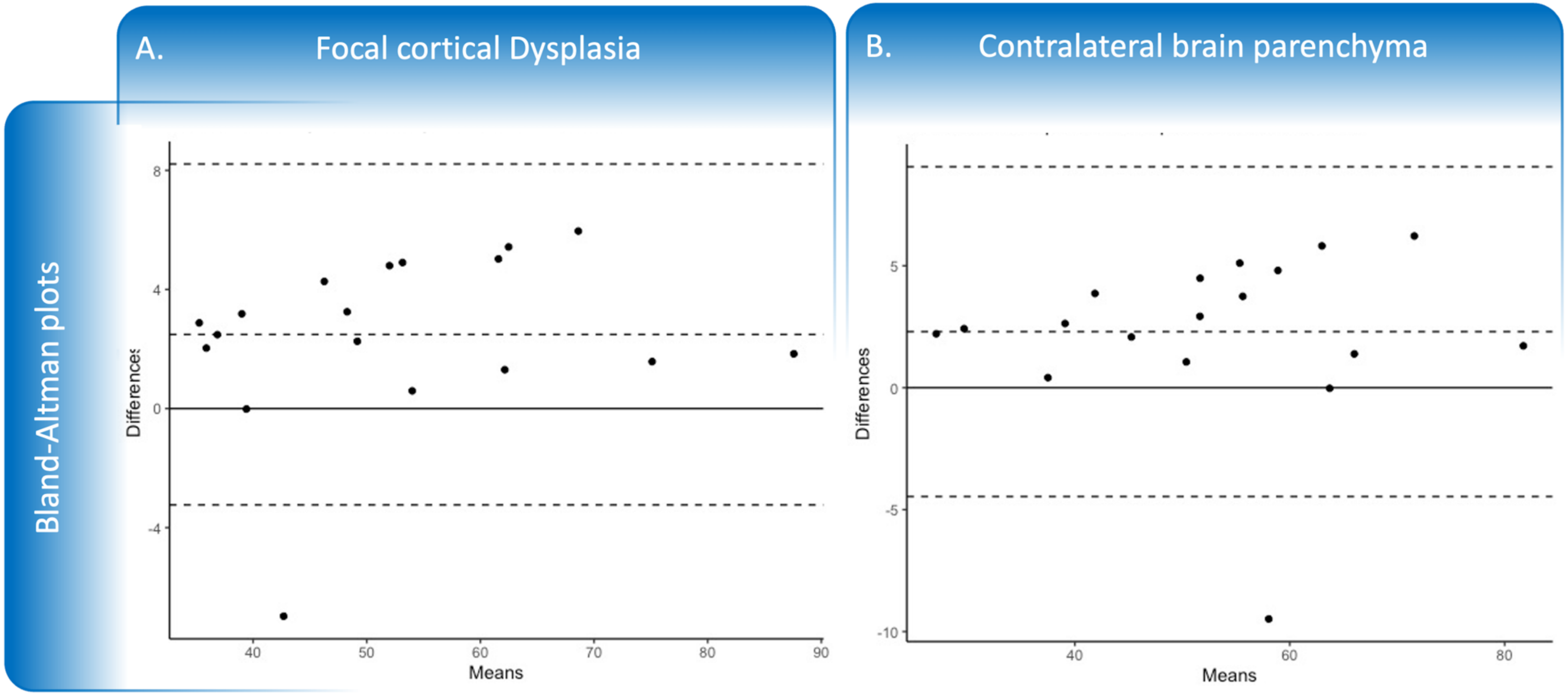

(A, B) Bland-Altman plots comparing the CBF and corrected values calculated in the focal cortical dysplasia (FCD, A) and the contralateral brain parenchyma (CBP, D). The mean difference and the confidence intervals were deemed within acceptable ranges.
